# Comparison of post-operative pain and quality of life between total thoracoscopic surgery and conventional full-sternotomy for aortic valve replacement

**DOI:** 10.1186/s12872-023-03617-w

**Published:** 2023-11-24

**Authors:** Zhiqin Lin, Xiujun Chen, Zheng Xu, Liangwan Chen, Xiaofu Dai

**Affiliations:** 1 Department of Cardiovascular Surgery, Union Hospital, Fujian Medical University, Xinquan Road 29#, Fuzhou, 350001 P. R. China; 2 Key Laboratory of Cardio-Thoracic Surgery, Fujian Medical University, Fujian Province University, Fuzhou, 350001 P. R. China

**Keywords:** Total thoracoscopic surgery, Aortic valve replacement, Pain, Quality of life

## Abstract

**Background:**

To compare the post-operative pain and quality of life of patients who underwent total thoracoscopic surgery (TTS) or conventional full-sternotomy (CFS) for aortic valve replacement (AVR).

**Methods:**

We reviewed the records of 223 consecutive AVR patients with either TTS or CFS from January 2018 to December 2022. We used a visual analogue scale (VAS) and the Short Form-36 Health Survey (SF-36) to measure the post-operative pain and quality of life, respectively. We also compared the operative data and clinical outcomes between the two groups.

**Results:**

The TTS group had lower adjusted mean VAS scores than the CFS group at all time points after surgery (at 1 to 3 days and at 3 and 6 months, *p* < .001 for all comparisons), indicating less pain. The TTS group also had higher mean SF-36 scores than the CFS group up to 6 months after surgery (*p* < .001 for all comparisons), indicating better quality of life. The operative time was similar between the two groups (*p* = .224), but the TTS group had longer cardiopulmonary bypass time and aortic cross-clamp time than the CFS group (*p* < .001). The TTS group had more pulmonary complications than the CFS group (*p* = .023). However, there were no significant differences in other major complications or mortality between the two groups.

**Conclusions:**

TTS is a safe and effective alternative to CFS for AVR. TTS resulted in less pain and better quality of life, especially in the early recovery period. However, further prospective randomized controlled studies are needed to confirm our findings.

## Background

Aortic valve replacement (AVR) surgery treats patients with symptomatic severe aortic stenosis or regurgitation [1, 2]. AVR can be performed using open-heart surgery or minimally invasive methods, such as transcatheter aortic valve replacement (TAVR) or total thoracoscopic surgery (TTS) [3]. TTS is a minimally invasive surgery that uses small incisions between the ribs, a video camera, and specialized instruments to access the heart. Compared to conventional full-sternotomy (CFS) AVR, TTS has several advantages, such as less blood loss, shorter hospital stay, faster recovery, and better cosmetic results [4, 5]. However, cardiac surgery poses a major challenge in managing postoperative pain, which affects the quality of life and outcomes of patients. Postoperative pain after surgery results from surgical trauma, inflammation, nerve damage, and chest tube insertion. Poorly controlled pain can impair pulmonary function, cause respiratory complications, chronic pain syndrome, and reduce patient satisfaction [6].

This study compared the postoperative pain and quality of life between TTS and CFS for AVR in a retrospective case series of 223 consecutive patients. We hypothesized that TTS would cause less postoperative pain and improve quality of life than FS.

## Materials and methods

### Study patients and data collection

This study is a retrospective analysis of metadata and clinical outcomes of patients who underwent isolated AVR by either TTS or CFS between January 2018 and December 2022 at a single center. The inclusion criteria were: (1) age ≥ 18 years; (2) symptomatic severe aortic valve disease, and indication for AVR according to the 2020 ACC/AHA guideline for the management of patients with valvular heart disease; (3) elective surgery. The exclusion criteria were: (1) emergency surgery; (2) previous cardiac surgery; (3) concomitant cardiac procedures. Demographic and clinical data were extracted from the medical records, including demographic information, preoperative risk factors, operative details, postoperative outcomes, complications, intensive care unit readmission rate and mortality.

The primary outcome measure was postoperative pain, assessed using the visual analogue scale (VAS) in numerical rating scales. The VAS is a 0–10 scale that patients use to rate their pain, where 0 means ‘no pain’ and 10 means ‘most pain’. Pain was assessed at 1 to 3 days and at 3 and 6 months after surgery. The secondary outcome measure was quality of life (QOL) as assessed by the Short Form-36 (SF-36) questionnaire, [7] which is a comprehensive 36-item survey that covers physical and mental health. We obtained permission from RAND Health Care to use the SF-36 survey in our research and followed their scoring instructions [8]. The items contribute to eight health domains: physical functioning, role limitations due to physical problems, bodily pain, general health, vitality, social functioning, role limitations due to emotional problems, and mental health. Each item is scored on a 0 to 100 range, where 100 represents the highest level of functioning possible. The items are then averaged together to create eight scale scores. Higher scores indicate better health status or functioning. The Physical Component Summary (PCS) scale is derived from the physical functioning, role limitations due to physical problems, bodily pain, and general health domains. The Mental Component Summary (MCS) scale is derived from the vitality, social functioning, role limitations due to emotional problems, and mental health domains. The SF-36 questionnaire was routinely administered by trained nurses to patients who underwent cardiac surgery at our center. The interviews were conducted at predefined time points: before surgery, 1 month, 3 months, 6 months, and 12 months after surgery. The data were collected and stored in a secure database with a quality control system to ensure accuracy and completeness.

The Ethics Committee of Union Hospital, Fujian Medical University approved this study, and all patient information will be kept confidential and secure. Informed consent was not required for this retrospective study.

### Surgical procedures and postsurgical treatment

All surgical procedures were performed under general anesthesia by experienced cardiovascular surgeons. The type of surgery was determined by the surgeon’s preference and experience, as well as the patient’s condition. There were no standardized selection criteria during the study period to determine which patients underwent TTS versus CFS. Patients with thoracic adhesions, chest wall deformity, or lung disease were excluded from thoracoscopic surgery because of difficulty in lung mobility.

TTS involved a 1 cm camera incision at the level of the anterior axillary line and a 3–4 cm main working port of anterior mini-thoracotomy in the third intercostal space. Peripheral cardiopulmonary bypass was established with vacuum-assisted double venous cannulation (internal jugular and femoral) and femoral arterial cannulation. The internal jugular vein was cannulated using a percutaneous Seldinger technique under echocardiographic guidance. A 2 cm incision was made in the right groin for femoral artery and vein cannulation. After establishing cardiopulmonary bypass, a Chitwood cross-clamp was inserted through the camera incision. Cold blood cardioplegia was delivered antegrade through a catheter inserted into the ascending aorta. The cross-clamp time was recorded as the interval between cross-clamp application and removal. CFS required a full sternotomy. The aortic valve was replaced with a bioprosthesis or mechanical prosthesis.

Postoperatively, all patients were transferred to the intensive care unit (ICU) for monitoring and management. They received standard postsurgical treatment for aortic valve replacement, including pain control, fluid management, and ventilator support. Pain control was similar between the two groups and was achieved using a multimodal approach, including nonsteroidal anti-inflammatory drugs, opioids, and regional anesthesia. All patients underwent transthoracic echocardiography (TTE) before hospital discharge and at follow-up visits.

### Statistical analysis

We expressed continuous variables as mean ± standard deviation or median (interquartile range [IQR]), depending on their distribution, and compared them using Student’s t-test or Wilcoxon signed-rank test, respectively. We presented categorical variables as counts or percentages and compared them using chi-squared test or Fisher’s exact test, as appropriate. To compare VAS scores and QOL outcomes across different time points and to assess the effect of surgical type, we fitted two types of generalized linear mixed-effects models for repeated-measures analysis. For VAS scores, we used a multinomial logistic model with an ordinal response variable. For QOL outcomes, we used a gaussian model with an identity link function. We treated differences among patients as a random effect and added “group*time” to the main effects with adjustment for baseline covariates. We excluded patients with missing data for a particular variable from the analysis of that variable. We fitted the models using the glmer function in the lme4 package and the clmm function in the ordinal package in R. We considered a two-sided *P*-value of < 0.05 as statistically significant. We performed all analyses using SPSS v.26.0 (IBM SPSS Inc., Armonk, NY) and R 4.0.1.

## Results

### Baseline characteristics

A total of 120 patients underwent thoracoscopic AVR and 103 underwent conventional sternotomy. The patients’ baseline characteristics and comorbidities are shown in Table 1. There were no significant differences between the two groups in terms of baseline demographic characteristics, comorbidities. The mean age of all patients was 57.0(51.5–64.0) years, and 59.2% were male. In this series, 22.4% of patients presented with aortic valve stenosis alone, 19.7% with aortic valve regurgitation alone and 57.8% with mixed stenotic and regurgitant lesions. A total of 164 (73.5%) patients had NYHA class III or IV.

**Table 1:** Comparison of patients’ baseline demographic and clinical characteristics

Variables^a^	Total sample (*n* = 223)	Patient groups		
		TTS (*n* = 120)	CFS (*n* = 103)	*P* value
Age, yr	57.0 (51.5–64.0)	57.0 (50.0–62.0)	57.0 (53.5–65.0)	0.302
Male, n (%)	132 (59.2%)	67 (55.88%)	65 (63.1%)	0.335
BMI, kg/m2	20.70 (18.65–24.65)	21.81 (18.65–24.65)	19.97 (18.43–22.95)	0.278
Smoking history, n (%)	56 (25.1%)	30 (25.0%)	26 (25.2%)	1.000
Diabetes, n (%)	36 (16.1%)	21 (17.5%)	15 (14.6%)	0.681
Hypertension, n (%)	53 (23.8%)	34 (28.3%)	19 (18.4%)	0.116
CAD, n (%)	36 (16.1%)	20 (16.7%)	16 (15.5%)	0.963
Prior MI, n (%)	8 (3.6%)	3 (2.5%)	5 (4.9%)	0.346
COPD, n (%)	12 (5.4%)	8 (6.7%)	4 (3.9%)	0.535
liver dysfunction, n (%)	21 (9.4%)	14 (10.0%)	7 (8.7%)	0.339
Dialysis, n (%)	12 (5.4%)	8 (6.7%)	4 (3.9%)	0.565
Peripheral Vascular Disease, n (%)	9 (4.0%)	4 (3.3%)	5 (4.9%)	0.565
Cancer history, n (%)	10 (4.5%)	7 (5.8%)	3 (2.9%)	0.293
Stroke history, n (%)	12 (5.4%)	7 (5.8%)	5 (4.9%)	0.980
History of AF, n (%)	10 (4.5%)	7 (5.8%)	3 (2.9%)	0.293
Endocarditis, n (%)	9 (4.0%)	5 (4.2%)	4 (3.9%)	0.915
NYHA class, n (%)				
I	41 (18.4%)	21 (17.5%)	20 (19.4%)	0.943
II	18 (8.1%)	10 (8.3%)	8 (7.8%)	
III	142 (63.7%)	76 (63.3%)	66 (64.1%)	
IV	22 (9.9%)	13 (10.8%)	9 (8.7%)	
Preoperative Aortic Valve Characteristics				
Simple Stenosis, n (%)	50 (22.4%)	27 (22.5%)	23 (22.3%)	0.845
Simple Insufficiency, n (%)	44 (19.7%)	22 (18.3%)	22 (21.4%)	
Mixed, n (%)	129 (57.8%)	71 (5.9%)	58 (5.6%)	
LVEF, %	53.9 (48.9–57.4)	55.3 (49.2-57.75)	52.9(48.4-57.225)	0.162

*Abbreviations: TTS *Total thoracoscopic surgery, *CFS *Conventional full-sternotomy, *BMI *Body mass index, *CAD* Coronary artery disease, *MI *Myocardial infarction, *COPD *Chronic obstructive pulmonary disease, *AF *Atrial fibrillation, *NYHA *New York Heart Association, *LVEF *Left ventricular ejection fraction

^a^Non-normally distributed variables are presented as the median [interquartile range (IQR)] and categorical data as number

### Operative data and early outcomes

Table 2 shows the operative data and early outcomes of the study participants. The total operative time was similar between the groups (TTS: 213(197-235.75) min vs. CFS: 208(195–224) min, *p* = .224). However, the TTS group had a significantly longer cardiopulmonary bypass time (TTS: 83.5(73.75-93) min vs. CFS: 62(56–64) min, *p* < .001) and aortic cross-clamp time (TTS: 56(52–62) min vs. CFS: 40(33–43) min, *p* < .001) compared to the CFS group. The groups differed significantly in respiratory complications (TTS: 12.5% vs. CFS: 25.2%, *p* = .023). Respiratory complications included prolonged intubation > 24 h, reintubation, pneumonia, acute respiratory distress syndrome, and pulmonary embolism. TTS patients also had a lower rate of prolonged ventilation time (≥ 24 h) (TTS: 12.5% vs. CFS: 25.2%, *p* = .039). However, there were no significant differences between the groups in other major complications, such as postoperative stroke, acute kidney injury, or low cardiac output syndrome. The TTS group had significantly shorter ICU stay and hospital stay than the CFS group (*p* < .001 for both comparisons). The overall hospital mortality was 2.7%, with two deaths (3.3%, both due to malignant arrhythmia) in the TTS group and four deaths (1.9%, one due to low cardiac output syndrome and multiple organ failure, one due to hematencephalon, and two due to severe pneumonia) in the CFS group (*p* = .522).

**Table 2:** Operative data and postoperative in-hospital outcomes

Variables^a^	Total sample (*n* = 223)	Patient groups		
		TTS (*n* = 120)	CFS (*n* = 103)	*P* value
Operation duration, minutes	210 (196.5–226)	213 (197-235.75)	208 (195–224)	0.224
CPB time, minutes	72 (61–86)	83.5 (73.75-93)	62 (56–64)	< 0.001
ACC time, minutes	50 (40–56)	56 (52–62)	40 (33–43)	< 0.001
Intensive care unit stay, days	2 (1–2)	1 (1–2)	2 (2–3)	< 0.001
Hospital stay, days	9 (8–11)	9 (8–10)	10 (9–12)	< 0.001
Hospital mortality, %	2.7%	3.3%	1.9%	0.522
Valve implantation				0.153
Bioprosthetic valve implantation, n (%)	54 (24.2%)	24 (20.0%)	35 (29.1%)	
Mechanical valve implantation, n (%)	169 (75.8%)	96 (80.0%)	68 (70.9%)	
Early complications				
Respiratory complication, n (%)	41 (18.4%)	15 (12.5%)	26 (25.2%)	0.023
Prolonged ventilation, n (%)	18 (8.1%)	5 (4.2%)	13 (12.6%)	0.039
LCOS requiring MCS, n (%)	2 (0.9%)	1 (0.8%)	1 (1.0%)	0.913
Cardiocerebral events, n (%)	9 (4.0%)	5 (4.2%)	4 (3.9%)	0.915

*Abbreviations: TTS *Total thoracoscopic surgery, *CFS* Conventional full-sternotomy, *CPB* Cardiopulmonary bypass, *ACC* Aortic cross-clamp, *LCOS* Low cardiac output syndrome, *MCS *Mechanical cardiac support

^a^Non-normally distributed variables are presented as the median [interquartile range (IQR)] and categorical data as number

### Post-operative pain and quality of life

**Fig. 1:**
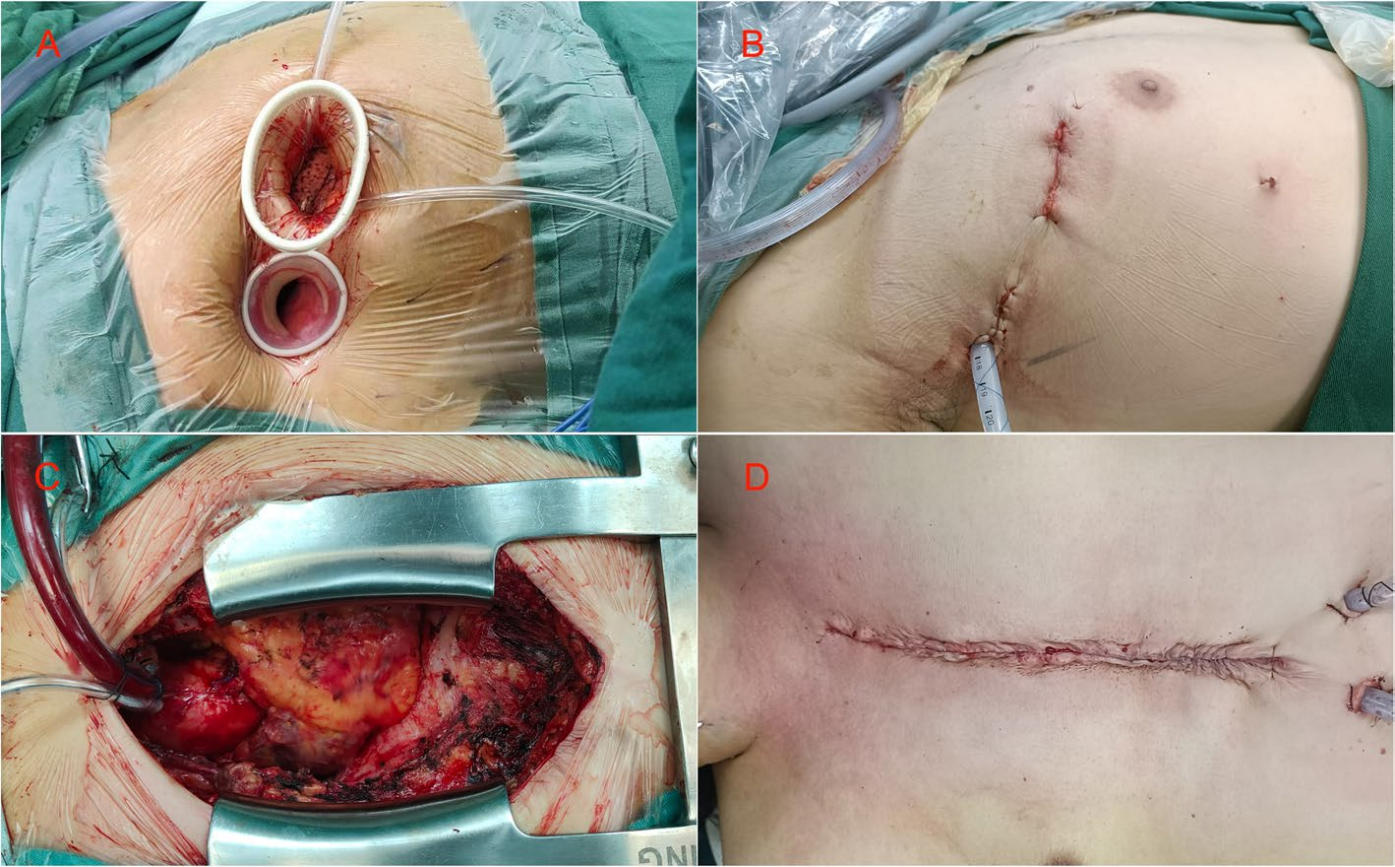
A comparison of the postoperative chest radiographs of a representative patient who underwent TTS (**A**, **B**) and a representative patient who underwent CFS (**C**, **D**), showing the difference of the surgical incisions and the chest tubes

**Fig. 2:**
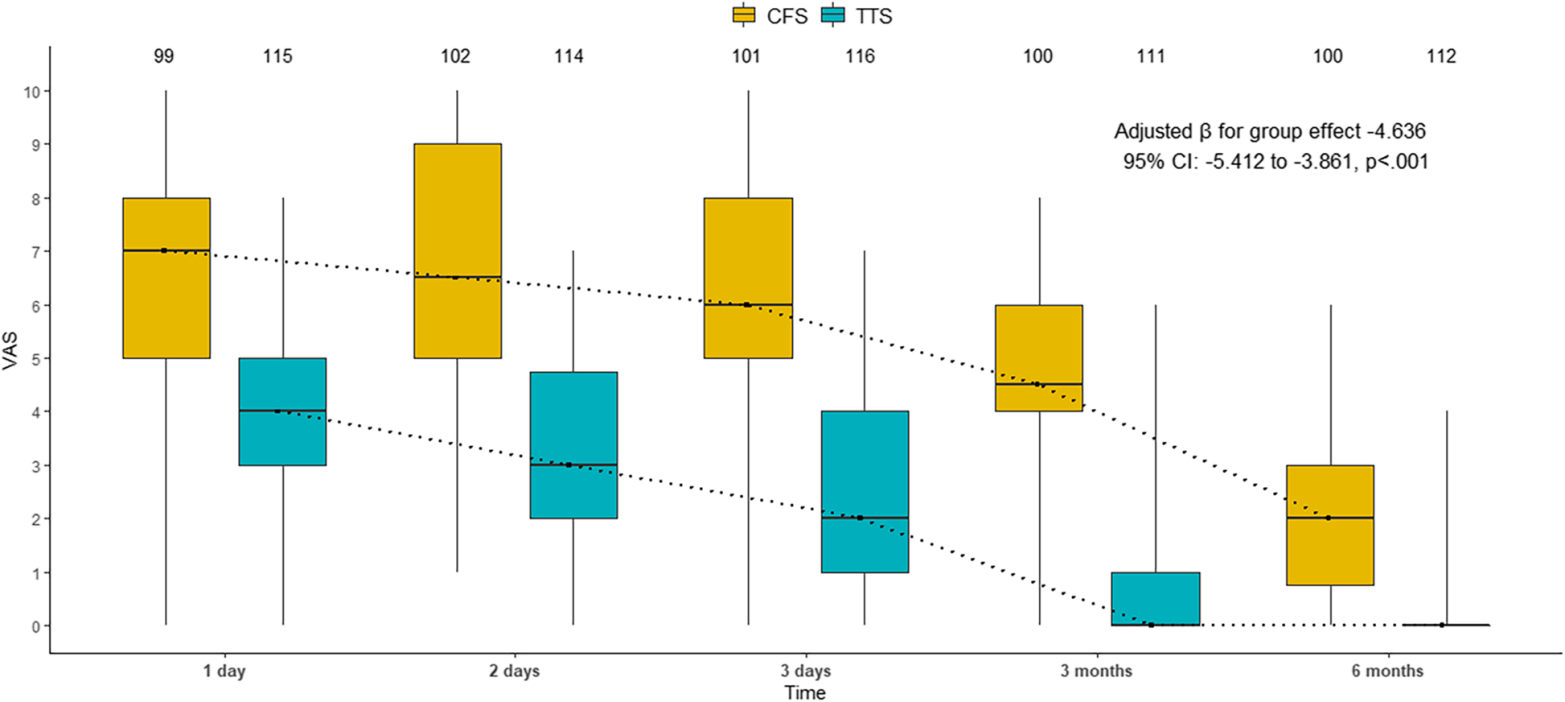
Median [interquartile range] of VAS of the two groups at follow-up. TTS, total thoracoscopic surgery; CFS, conventional full-sternotomy; VAS, visual analogue scales

**Fig. 3:**
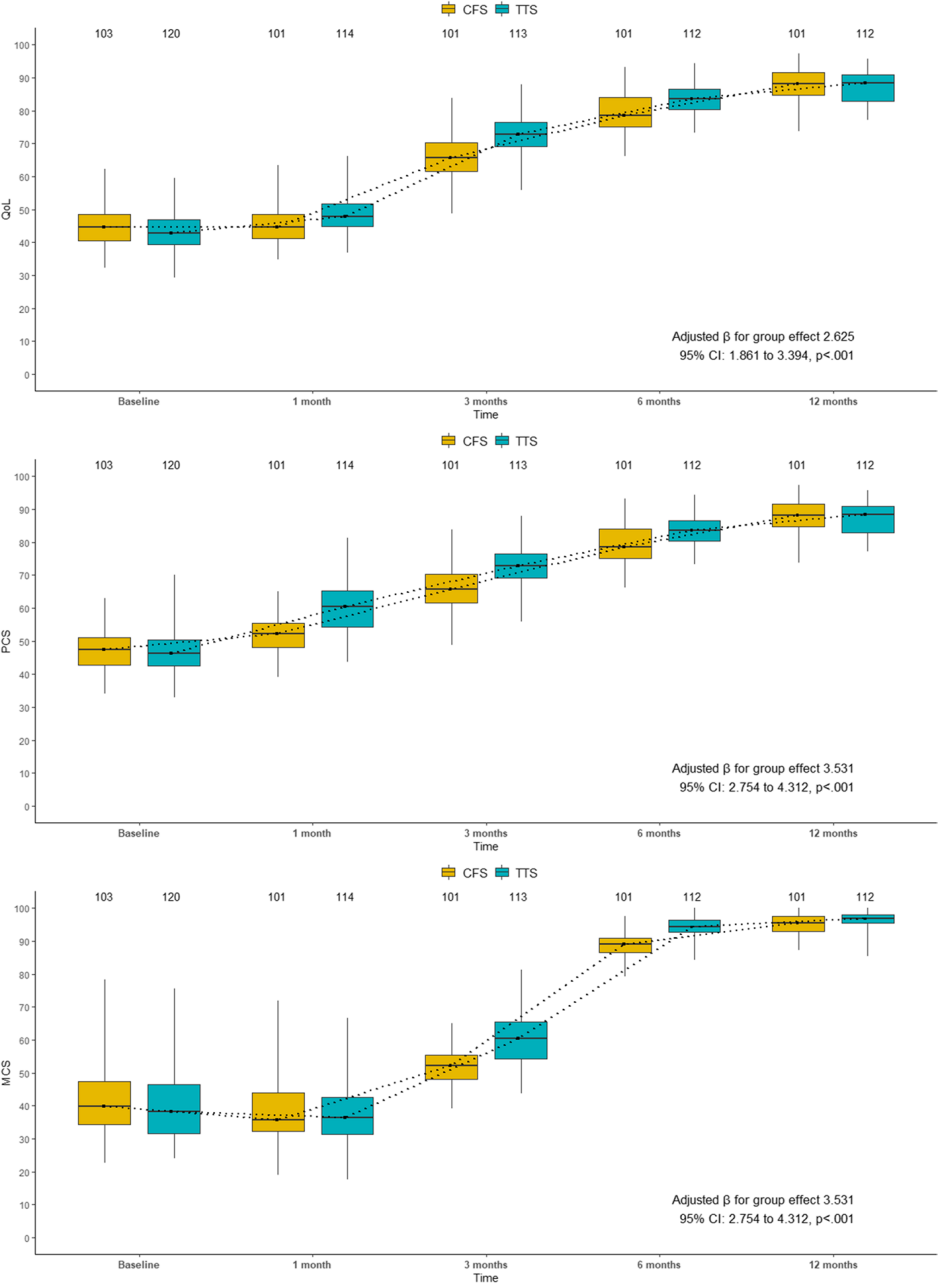
Median [interquartile range] of QOL, MCS, and PCS of the two groups at baseline and follow-up. TTS, total thoracoscopic surgery; CFS, conventional full-sternotomy; QOL, quality of life; PCS, physical component summary; MCS: mental component summary

Figure 1A-D shows the difference of the surgical wounds and the chest tubes. The TTS group had smaller and less visible wounds than the CFS group, and the chest tubes were inserted through the camera incision in the TTS group, while they were inserted through two separate incisions in the CFS group. We analyzed the VAS scores and SF-36 scores using generalized linear mixed models. These models can account for the repeated measures and the random effects of the patients. However, for clarity and completeness, we also present the point by point comparison of pain and QoL between the two groups at each time point. These details are shown in Table 3 and Figs. 2 and 3. The adjusted mean VAS score decreases over time for both groups (*p* < .001 for all comparisons), but more sharply for TTS patients than FS patients within 3 months of surgery (*p* < .001). The adjusted mean VAS score is also lower for TTS group than FS group at each time point (*p* < .001 for all comparisons). The treatment groups had comparable adjusted mean baseline scores for QOL, PCS and MCS (*p* > .05 for all comparisons). At 1month post-operation, the adjusted mean scores for QOL and PCS were significantly higher for TTS patients than for CFS patients (*p* < .001 for both comparisons), while the adjusted mean score for MCS was similar between the groups (*p* = .326). At 3 months post-operation, the adjusted mean scores for all three outcomes were significantly higher for TTS patients than for CFS patients (*p* < .001 for all comparisons). At 6 months post-operation, the same pattern was observed (*p* < .001 for all comparisons). At 12 months post-operation, the treatment groups had similar adjusted mean scores for all three outcomes (*p* > .05 for all comparisons).

**Table 3:** Summary of mixed-model repeated measures analysis for VAS scores and SF-36 scores

Response variables	Thoracoscopy		Conventional		Difference (95% CI; *p* value)
	*n*	Adjusted mean	*n*	Adjusted mean	
VAS score^a^					
Day 1	115	-1.250	99	2.540	3.790 (3.120~4.470; *p* = .433)
Day 2	114	-2.700	102	2.100	4.790 (4.110~5.470; *p* < .001)
Day 3	116	-3.970	101	1.470	5.450(4.750~6.150; *p* < .001)
Month 3	111	-6.770	100	-1.030	5.740 (4.970~6.510; *p* < .001)
Month 6	112	-8.930	100	-5.480	3.450 (2.550~4.350; *p* < .001)
QOL score^b^					
Baseline	120	45.1	103	45.6	0.554 (-0.836~1.940; *p* = .434)
Month 1	114	50.2	101	46.7	-3.534 (-4.947~-2.120; *p* < .001)
Month 3	113	74.1	101	67.5	-6.654 (-8.070~-5.240; *p* < .001)
Month 6	112	84.8	101	80.6	-4.125 (-5.545~-2.710; *p* < .001)
Month 12	112	88.6	101	89.1	0.495 (-0.924~1.910; *p* = .494)
PCS score^c^					
Baseline	120	47.9	103	48.3	0.465 (-1.020~1.950; *p* = .539)
Month 1	114	61.1	101	53.0	-8.024 (-9.530~-6.510; *p* < .001)
Month 3	113	73.7	101	67.1	-6.636 (-8.150~-5.120; *p* < .001)
Month 6	112	84.3	101	80.2	-4.113 (-5.630~-2.600; *p* < .001)
Month 12	112	88.2	101	88.7	0.506 (-1.010~2.020; *p* = .513)
MCS score^d^					
Baseline	120	40.1	103	40.7	0.656 (-1.120~2.432; *p* = .469)
Month 1	114	37.2	101	38.6	0.906 (-0.901~2.712; *p* = .326)
Month 3	113	59.9	101	51.6	-8.224 (-10.034~-6.413; *p* < .001)
Month 6	112	94.0	101	88.1	-5.847 (-7.661~-4.032; *p* < .001)
Month 12	112	96.1	101	94.9	-1.223 (-3.037~0.592; *p* = .187)

*Abbreviations: VAS *Visual analogue scales, *SF-36 *Short Form-36, *QOL *Quality of life, *PCS *Physical component summary, *MCS *Mental component summary, *CI *Confidence intervals

^a^Adjusted for baseline covariates in a multinomial logistic model with an ordinal response variable: age, gender

^b^Adjusted for baseline covariates in a gaussian model with an identity link function: age, gender, diabetes, hypertension, peripheral vascular disease, hypohepatia, COPD, Cancer, stroke/TIA, CAD, liver dysfunction, baseline QOL score

^c^Adjusted for baseline covariates in a gaussian model with an identity link function: age, gender, diabetes, hypertension, peripheral vascular disease, hypohepatia, COPD, Cancer, stroke/TIA, CAD, liver dysfunction, baseline PCS score

^d^Adjusted for baseline covariates in a gaussian model with an identity link function: age, gender, diabetes, hypertension, peripheral vascular disease, hypohepatia, COPD, Cancer, stroke/TIA, CAD, liver dysfunction, baseline MCS score

## Discussion

This retrospective comparative study analyzed the post-operative pain and QOL between TTS and CFS for AVR. Our findings indicate that TTS for AVR is associated with lower post-operative pain and higher QOL measures compared to CFS for AVR in the early post-operative period.

The advantages of minimally invasive TTS over CFS have been well documented in previous studies [9]. Minimally invasive TTS can reduce surgical trauma, blood loss, wound infection, postoperative pain, and hospital stay, as well as improve cosmetic results and patient satisfaction [10, 11]. Thoracoscopic surgery can also preserve the integrity of the chest wall and reduce postoperative pain and analgesic consumption. Moreover, TTS can provide a clear and magnified view of the operative field through a video camera, which may facilitate precise manipulation and suturing [12].

TTS also has some drawbacks and limitations. First, TTS requires a longer cardiopulmonary bypass time and aortic cross-clamp time than CFS, [13] which may increase the risk of ischemia-reperfusion injury and neurological complications [14]. Second, TTS requires specialized instruments and skills, as well as a steep learning curve for surgeons [15]. Third, complex aortic valve conditions, such as severe calcification of the aortic annulus or small aortic annulus diameter that necessitates annulus enlargement, may pose challenges for thoracoscopic AVR [13].

Therefore, careful patient selection and surgical experience are essential for successful TTS [16, 17].

In this study, we performed two-port thoracoscopic AVR for patients with aortic valve disease who met the inclusion and exclusion criteria. We found that TTS was feasible and safe in these patients. The operative time was similar between the two groups, indicating that thoracoscopic surgery did not prolong the overall duration of surgery. However, the cardiopulmonary bypass time and aortic cross-clamp time were significantly longer in the thoracoscopic group than in the sternotomy group. This was consistent with previous studies that reported longer cardiopulmonary bypass time and aortic cross-clamp time for thoracoscopic AVR compared to CFS [13, 18, 19]. TTS requires longer cardiopulmonary bypass and aortic cross-clamp times because of technical challenges. These include limited exposure, restricted mobility, lack of tactile feedback, and need for precise coordination. Improving surgical techniques and instruments may help to shorten these times. In this study, we utilized interrupted pledgeted mattress sutures for implantation of the bioprosthetic or mechanical valves. The sutures were tied down manually after knot pushing and securing with a surgical clip applier. We did not employ knot tying devices such as the Kor-Knot or sutureless valve technology in this series. Use of automated knot tying tools or rapid deployment valves may help shorten the cross clamp and cardiopulmonary bypass times compared to manual tying techniques. However, manual tying allows for fine control of valve orientation and seating during implantation. Further comparative studies are warranted to determine if knot tying devices or sutureless valves provide advantages over manual techniques for total thoracoscopic aortic valve replacement, especially with regards to facilitating reduced cross clamp times. Our center will be investigating these technologies in the future to potentially optimize our total thoracoscopic approach.

The main benefit of TTS was the reduction of postoperative pain. We found that the VAS scores were significantly lower in the TTS group than in the CFS group at all time points after surgery. This indicated that TTS caused less surgical trauma and inflammation than CFS. Moreover, TTS avoided sternal retraction and division, which may damage the intercostal nerves and cause chronic pain syndrome. The reduction of postoperative pain may have several positive effects on patient recovery and outcomes. First, less postoperative pain may improve pulmonary function and prevent respiratory complications [20]. Second, less postoperative pain may reduce analgesic consumption and its related side effects [21]. Third, less postoperative pain may enhance patient comfort and satisfaction [22]. The difference in VAS scores between the two groups was most prominent within 3 months after surgery, indicating that the pain-relieving benefits of TTS were greatest during the early recovery period. These results are supported by previous studies demonstrating less postoperative pain in TTS relative to CFS patients, especially in the first few months following surgery [13, 16].

Another benefit of thoracoscopic surgery was the improvement of QOL. We found that the QOL scores were significantly higher in the thoracoscopic group than in the sternotomy group up to 6 months after surgery. This indicated that thoracoscopic surgery had a positive impact on the physical and mental well-being of the patients. The QOL scores were measured by the SF-36 questionnaire, which covers eight domains of health status. We found that the thoracoscopic group had better scores than the sternotomy group in most domains, especially in physical functioning, role limitations due to physical problems, bodily pain, and general health. These domains reflect the ability of the patients to perform daily activities, cope with physical challenges, and enjoy life without pain or discomfort. The improvement of these domains may be related to the reduction of postoperative pain, faster recovery, and better cosmetic results after TTS. However, we also found that the QOL scores were similar between the two groups at 12 months after surgery. This suggested that the benefits of TTS on QOL were not sustained in the long term. This may be explained by the fact that QOL is influenced by many factors besides surgery, such as age, comorbidities, social support, and lifestyle [23]. Moreover, QOL may also depend on the type and durability of the prosthetic valve used for AVR [24]. Therefore, further studies with longer follow-up and larger sample size are needed to evaluate the long-term effects of TTS on QOL.

This study has several limitations that may affect the generalizability and validity of our findings. First, it is a retrospective, single-center study with a relatively small sample size. Second, we did not compare TTS with other minimally invasive approaches for AVR, such as TAVR or partial sternotomy. These approaches may have different advantages and disadvantages in terms of safety, efficacy, quality of life, and cost, which are important considerations when determining the most appropriate surgical option for patients. Therefore, further studies are needed to compare thoracoscopic surgery with TAVR or partial sternotomy. Third, a lack of randomization between surgical techniques and potential surgeon selection bias regarding operative approach limit the study. We also did not assess long-term outcomes, such as survival and valve durability, which are important considerations in AVR.

## Conclusion

In this retrospective study comparing 223 patients who underwent TTS versus CFS for AVR, we found that TTS was associated with significantly lower postoperative pain scores and higher quality of life scores in the first 6 months after surgery. There were no significant differences in operative time, mortality, or most major complications between the groups. However, due to the non-randomized, retrospective design, we cannot make definitive conclusions about the comparative safety or efficacy of TTS versus CFS. Additional prospective, randomized studies are needed to better understand the risk-benefit profile of TTS for AVR. The potential advantages of TTS observed in this initial study, including less postoperative pain and improved quality of life, merit further investigation in controlled trials comparing TTS to conventional sternotomy as well as other minimally invasive approaches.

## Data Availability

All data generated or analyzed during this study are included in this published article.

## Abbreviations

AVR: Aortic valve replacement
TAVR: Transcatheter aortic valve replacement
TTS: Total thoracoscopic surgery
CFS: Conventional full-sternotomy
VAS: Visual analogue scale
QOL: Quality of life
SF-36: Short Form-36
PCS: Physical Component Summary
MCS: Mental Component Summary
ICU: Intensive care unit
TTE: Transthoracic echocardiography
IQR: Interquartile range
